# White Paper: Mimetics of Class 2 Tumor Suppressor Proteins as Novel Drug Candidates for Personalized Cancer Therapy

**DOI:** 10.3390/cancers14184386

**Published:** 2022-09-09

**Authors:** Edgar Dahl, Sophia Villwock, Peter Habenberger, Axel Choidas, Michael Rose, Bert M. Klebl

**Affiliations:** 1 Institute of Pathology, Medical Faculty, RWTH Aachen University, D-52074 Aachen, Germany; 2 Center for Integrated Oncology Aachen Bonn Cologne Duesseldorf (CIO ABCD), D-52074 Aachen, Germany; 3 Lead Discovery Center GmbH (LDC), Otto-Hahn-Straße 15, D-44227 Dortmund, Germany

**Keywords:** tumor suppressor proteins, mimetics, personalized cancer therapy, class 2 tumor suppressor genes, DNA methylation drivers, SFRP1, ITIH5, small molecules

## Abstract

**Simple Summary:**

A concept is presented for a new therapeutic approach, still in its early stages, which focuses on the phenotypic mimicry (“mimesis”) of proteins encoded by highly disease-relevant class 2 tumor suppressor genes that are silenced by DNA promoter methylation. Proteins derived from tumor suppressor genes are usually considered control systems of cells against oncogenic properties. Thus they represent the brakes in the “car-of-life.” Restoring this “brake function” in tumors by administering mimetic drugs may have a significant therapeutic effect. The proposed approach could thus open up a new, hitherto unexploited area of research for the development of anticancer drugs for difficult-to-treat cancers.

**Abstract:**

The aim of our proposed concept is to find new target structures for combating cancers with unmet medical needs. This, unfortunately, still applies to the majority of the clinically most relevant tumor entities such as, for example, liver cancer, pancreatic cancer, and many others. Current target structures almost all belong to the class of oncogenic proteins caused by tumor-specific genetic alterations, such as activating mutations, gene fusions, or gene amplifications, often referred to as cancer “driver alterations” or just “drivers.” However, restoring the lost function of tumor suppressor genes (TSGs) could also be a valid approach to treating cancer. TSG-derived proteins are usually considered as control systems of cells against oncogenic properties; thus, they represent the brakes in the “car-of-life.” Restoring these tumor-defective brakes by gene therapy has not been successful so far, with a few exceptions. It can be assumed that most TSGs are not being inactivated by genetic alteration (class 1 TSGs) but rather by epigenetic silencing (class 2 TSGs or short “C2TSGs”). Reactivation of C2TSGs in cancer therapy is being addressed by the use of DNA demethylating agents and histone deacetylase inhibitors which act on the whole cancer cell genome. These epigenetic therapies have neither been particularly successful, probably because they are “shotgun” approaches that, although acting on C2TSGs, may also reactivate epigenetically silenced oncogenic sequences in the genome. Thus, new strategies are needed to exploit the therapeutic potential of C2TSGs, which have also been named DNA methylation cancer driver genes or “DNAme drivers” recently. Here we present a concept for a new translational and therapeutic approach that focuses on the phenotypic imitation (“mimesis”) of proteins encoded by highly disease-relevant C2TSGs/DNAme drivers. Molecular knowledge on C2TSGs is used in two complementary approaches having the translational concept of defining mimetic drugs in common: First, a concept is presented how truncated and/or genetically engineered C2TSG proteins, consisting solely of domains with defined tumor suppressive function can be developed as biologicals. Second, a method is described for identifying small molecules that can mimic the effect of the C2TSG protein lost in the cancer cell. Both approaches should open up a new, previously untapped discovery space for anticancer drugs.

## 1. Problem to Be Solved

### 1.1. Current Personalized Cancer Therapy Is Dependent on the Presence of Druggable Driver Alterations, and Therefore Its Use Is Limited in Some Tumor Entities

In its search for new target molecules to combat cancer, the pharmaceutical industry has so far mainly focused on oncogenic proteins encoded by genetic driver alterations. These cancer-promoting proteins are either activated at the molecular level, e.g., through tumor-specific amino acid changes [1] or represent fusion proteins able to promote ligand-independent signaling [2] or are strongly overexpressed due to amplification [3]. Overexpression and/or molecular alteration of these proteins often lead to permanent activation of oncogenic signaling pathways that promote tumor development and disease progression. Thus, direct inhibition of these oncogenic proteins by either therapeutic antibodies or small molecules is widely used in personalized cancer therapy to suppress tumor growth. Though this concept has been successful and still is a very valid approach for cancer drug development, it nevertheless has certain limitations:

(1) There are tumor diseases in which virtually no druggable genetic driver alterations have been found so far, despite intensive research. Prostate cancer [4] and liver cancer [5] are such examples, their genetic landscapes presenting considerably less druggable alterations compared with, e.g., lung or colon cancer.
(2) Even in tumors with many known druggable driver mutations, there are histological subgroups that have significantly fewer druggable driver alterations, for example, small cell lung cancer (SCLC) [6] compared with non-small cell lung cancer (NSCLC) or within NSCLC, squamous cell carcinoma (SqCC) [7] compared with adenocarcinoma (AC). Many histological subgroups of important cancer entities are, therefore, not sufficiently druggable by inhibiting genetic driver alterations.
(3) Driver alterations are often referred to as the “Achilles’ heel” [8] in combating the tumor, as the growth of the affected cancer tissue is usually heavily dependent on the metabolic route activated by the driver alteration, a status called oncogene addiction [9]. On the one hand, this is a very helpful property because, in principle, it should thus be possible to develop targeted therapy drugs with few side effects. On the other hand, however, it has repeatedly been observed that targeted therapies completely fail after an excellent initial response, often within a few months of therapy, because resistance quickly develops. A prominent example is the reappearance of large piles of melanoma tumors after therapy resistance occurs to BRAF inhibitors targeting the V600E mutation [10]. In melanoma and other tumor entities, a rapid selection for cancer cells arise that bypass the effectiveness of the targeted therapy. Well-known examples are the RAS-activating mutations in BRAF actionable melanoma or the T790M resistance mutation in non-small cell lung cancer (NSCLC) under therapy with first or second generations tyrosine kinase inhibitors (TKIs) directed against the EGFR receptor [11].

### 1.2. Cancer Gene Therapy Is Suitable to Lift the Potential of Tumor Suppressor Genes, but Its Success Has Been Hampered by Low Efficiency of Gene Delivery

In principle, gene therapy may be used to reactivate both class 1 and class 2 tumor suppressor genes that have become mutated or lost in the tumor. Though this concept has been pursued for more than 25 years now, it has only been moderately successful [12]. Although gene therapy has recently become successful in directly correcting monogenic disorders [13], this success has not yet been translated to tumors. The reason is the low efficiency of gene delivery, which largely is irrelevant in the clinical correction of monogenic disorders—in some cases, less than 10% of the normal concentration of the protein can restore health. In the treatment of cancers, such low uptake of corrective gene transfer into the tumor tissue likely is insufficient with regard to disease progression. As of today, most gene transfer vectors have inadequate infectivity or a limited biodistribution to transfer the desired corrective genes to just about any cancer cell. Still, there are a few examples where cancer gene therapy was successful. An example is Gendicine, which uses a recombinant human p53 adenovirus [14]. This drug, developed by a Chinese gene therapy company, was approved in 2003 by the China Food and Drug Administration (CFDA) as a gene therapy product to treat head and neck cancer. Gendicine is delivered via minimally invasive intratumoral injection, as well as by intracavity or intravascular infusion. The wild-type p53 protein expressed by Gendicine-transduced cancer cells is able to mediate cell-cycle arrest and induce apoptosis. Gendicine has exhibited a good safety record and, when combined with chemotherapy and radiotherapy, has demonstrated significantly higher response rates than standard therapy [14].

### 1.3. Genome-Wide Epigenetic Therapies May Reactivate Key Class 2 Tumor Suppressor Genes, but the Interplay of Molecular Pathways Affected Is Hard to Control

The cancer epigenomes of all tumor entities examined so far exhibit both stochastic and characteristic changes in global DNA methylation, such as the promoter DNA hypermethylation of tumor suppressor genes and hypomethylation in other genomic regions such as repetitive elements [15,16,17]. Unlike class 1 tumor suppressor genes, which are being inactivated by permanent DNA alterations (mutation, chromosomal deletion), C2TSGs are inactivated by epigenetic processes that lead to loss of expression and are reversible, in principle. C2TSGs were already defined by Ruth Sager and coworkers in the 1990s [18,19]. Epigenetic drugs such as DNA methyltransferase inhibitors (demethylating agents) or histone deacetylase inhibitors are thought to induce their tumor suppressive properties due to the reactivation of epigenetically silenced C2TSGs. This approach has been partially successful in hematologic malignancies but, so far, not in solid tumors [20]. DNA methylation inhibitors azacytidine and decitabine were approved by the FDA in 2004 and 2006, respectively, for the treatment of patients with myelodysplastic syndromes and HDAC inhibitors such as Vorinostat (approved in 2006) for the treatment of patients with recurrent cutaneous T-cell lymphoma. Despite this initial promise in liquid cancers, for solid tumors, no epigenetic drugs were approved in the 2010s [21]. This may be due to the fact that these rather global epigenetic therapies are shotgun approaches, acting both on tumor suppressor genes and silenced oncogenic sequences in the genome, such as human endogenous retroviruses (ERVs) and other transposable elements, which are usually suppressed by DNA methylation in somatic cells [22]. In addition, several technical problems have arisen when trying to quantify the effect of epigenetic drugs in solid tumors since it is difficult to decide what, when, and where to measure [21]. Concerning demethylating agents, the effect was usually measured by evaluating global DNA methylation, while other authors determined the methylation status of specific genes such as GADD45A [23]. From our own experience in epigenetic cancer research, we can report that even in cell culture and thus under optimal conditions, not all the C2TSGs can be reactivated to the same extent by demethylating agents. There are major differences between different C2TSGs in their reactivability, and the causes are still poorly understood. In summary, one may even argue that genome-wide epigenetic therapies do not quite match the therapeutic requirements of the era of personalized therapy. Thus, our focus should be on developing approaches to specifically reactivate key genes, which are epigenetically silenced, i.e., validated C2TSGs or DNAme drivers.

## 2. Our Proposed Solution to the Problem: Specific C2TSGs Mimetics

In human cancers of nearly all tumor entities, a shutdown of tumor suppressive signaling pathways by C2TSG silencing occurs much more frequently and also usually earlier in the disease process than activation of oncogenic signaling pathways by typical genetic driver alterations [15,16,17]. Indeed, both epigenetics and genetic alterations may constitute pivotal drivers of early cancer development, and their contribution in these early steps of the disease may differ dependent on the tumor entity under investigation. The epigenetic contribution has been particularly well studied in recent work on field changes in DNA methylation during bladder cancer development [24]. It was concluded that DNA methylation might serve as an initiator of carcinogenesis that precedes DNA copy alterations and driver gene mutations. It was suggested that these later genetic alterations might reinforce oncogenic mechanisms already initiated by DNA methylation.

Within the multistep process of epigenetic silencing, tumor-specific promoter DNA methylation of C2TSGs [16] is considered the last and best tangible step leading to permanent transcriptional shutdown and thus to loss of tumor suppressor protein and function. Although it seems natural to assume that all observed promoter DNA methylation changes deterministically occur and drive the cancer phenotype, in vitro models and data from human cancers have shown that most DNA methylation changes follow a stochastic process [25]. Thus, it is not possible to derive from the frequency of promoter DNA methylation in the tumor whether a silenced gene is associated with the cancer phenotype or not. This situation is reminiscent of the challenge of distinguishing driver and passenger mutations in the search for druggable oncogenes. Therefore, a variety of bioinformatic methods have been developed to identify DNA methylation cancer driver genes [26,27,28]. New studies combine these approaches with “state of the art” cell biological approaches such as CRISPR knock-outs for validation of DNA methylation driver genes, or short named “DNAme drivers” [28]. In this white paper, we continue to call DNAme drivers as C2TSGs in recognition of the original hypothesis formulated by Ruth Sager [18].

**Figure 1:**
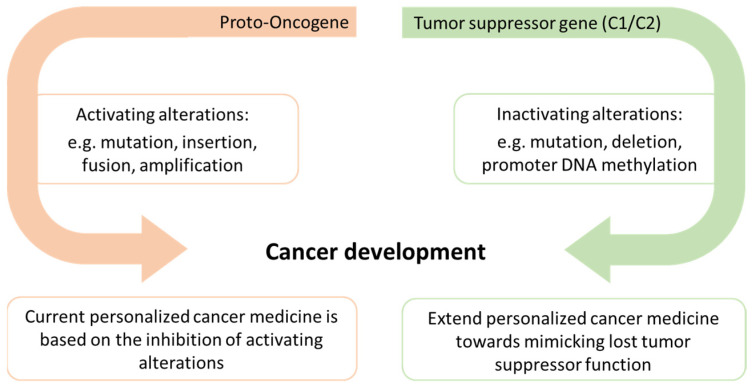
Alterations in both proto-oncogenes and tumor suppressor genes may lead to cancer development, but current personalized cancer medicine is mainly based on the inhibition of activating alterations. We propose to expand the scope of personalized cancer medicine by developing tumor suppressor protein-specific mimetic drugs that can partially or fully substitute the lost tumor suppressive function.

A recent landmark publication has demonstrated that there are probably several hundreds of C2TSGs silenced in each tumor entity [28]. The numbers ranged from 189 in chronic lymphocytic leukemia (CLL) to 503 in breast cancer (high-grade ductal carcinoma in situ). However, the significance of their loss for tumor initiation and progression is still insufficiently understood for most C2TSGs. Achieving insights into C2TSG function in vitro and in vivo thus offers a large space for new therapeutic approaches. Here we propose to foster a new field of personalized cancer medicine by developing drugs that mimic lost tumor suppressor protein functions (Figure 1).

**Figure 2:**
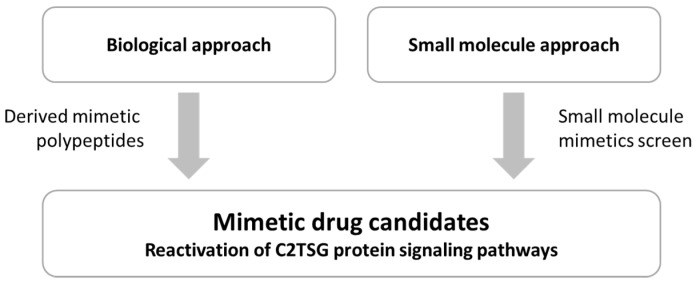
Biological and small molecule approach toward defining mimetic drug candidates able to reactivate the signaling pathway normally controlled by the C2TSG protein. Restoration of these growth suppressive signaling pathways should have a significant inhibitory effect on tumor initiation and progression.

These mimetic drug candidates or more simple “mimetics” can, for example, be truncated, genetically improved, or more stable variants of the tumor suppressor protein itself (biological approach) or small molecules which mimic the function of the lost tumor suppressor protein. Both translational approaches are expected to reactivate the C2TSG protein signaling pathway suppressed in the tumor (Figure 2), thus causing a therapeutic effect.

Mimetic drugs are well known in the pharmaceutical industry, but their meaning is somewhat different from the C2TSG protein mimetics concept we propose in this white paper. In the pharmaceutical industry, both “peptidomimetics” drugs [29], i.e., small polypeptide chains designed to mimic larger polypeptides and “small molecule mimetics,” such as the BH3 mimetics [30] that mimic the interaction of a protein (BH3) with an (anti-apoptotic) partner protein, have been developed. Our definition of a mimetic drug imitating or mimicking a C2TSG protein is more generic, as it includes from the compound site, both polypeptides and small molecules, and from the mimicking site, both the C2TSG protein itself and other key molecules in the C2TSG signaling pathway, which forward the C2TSG signal.

## 3. Two Straightforward Approaches to Develop C2TSG Specific Mimetic Drugs

We present a platform technology for the identification of C2TSG mimetics (of both biological and small molecule types), which we have developed over the last years. Of course, both strategies (i.e., biological and small molecule approach) rely on the identification and validation of suitable C2TSG candidates, which will be further explored and prioritized for a mimetic drug development project. To this end, our research group recently has developed a comprehensive strategy to identify novel C2TSGs based on a consecutive analysis of expression, methylation, and survival data using the TCGA data set [31,32], but also has relied on C2TSG candidates previously found by other screening approaches [33,34]. As these approaches usually deliver many hundreds of putative C2TSG, several filtering steps are needed to select a handful of leading C2TSGs for later prioritization. First, a clear correlation between promoter methylation of the C2TSG candidate and loss of expression must be shown. This does not generally hold true for tumor suppressor genes characterized so far, some of which can undergo both genetic and epigenetic alterations. Next, a strong correlation between promoter methylation on the one side and unfavorable prognosis on the other is mandatory to consider a C2TSG as a tumor suppressor gene candidate [35]. In addition, information derived from extensive database studies on the complexity of C2TSG expression (number of transcripts, alternative splicing, or alternative promoters) and the cellular localization and topology of the deduced protein can be used to select favorites.

Particularly interesting C2TSGs, such as those encoding the extracellular matrix protein ITIH5 [35,36] or the Wnt antagonist SFRP1 [37,38], are presented as “use cases” in this white paper. Extensive validation has been carried out demonstrating their C2TSG properties. This includes expression and methylation analysis both in a variety of tumor cell lines and in large tumor patient cohorts, but also in-depth analysis of forced C2TSG re-expression in human cancer cell lines and loss of expression in transgenic mouse models [39,40]. Further knowledge on putative signaling pathways and protein domain function has been generated to better understand the biology that enables the tumor suppressive function measured in vitro or in vivo. Correlation between C2TSG protein expression and patient survival data is usually achieved by analyzing large cohorts of tumors assembled on tissue microarrays by immunohistochemistry. In summary, all these experimental data on novel C2TSGs and their biology can be combined to prioritize certain C2TSG candidates for a novel translational mimetic drug development project.

### 3.1. The Biological Approach Defines Key Tumor Suppressive Domains in a C2TSG Protein

**Figure 3:**
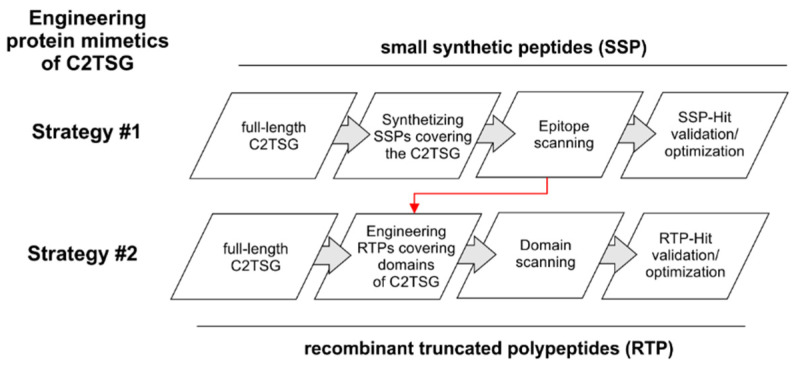
Schematic workflow to present two different strategies to engineer protein mimetics of C2TSG proteins, simulating/restoring their tumor suppressive function. Strategy #1 involves a systematic epitope scanning using partially overlapping chemically synthesized peptides covering the whole C2TSG protein to highlight hit regions. Strategy #2 follows the biological protein structure considering distinct protein domains. On this basis, truncated polypeptides are engineered and synthesized in vitro. Red arrow: data of hit regions of the epitope mapping study could be considered for prioritization of domains in Strategy #2.

In the last decades, peptides, proteins, and other biotechnologically derived products have been shown to be promising candidate compounds in the fight against cancer [41]. The here described biological approach pursues the identification of amino acid sequences of full-length C2TSG proteins suitable for engineering polypeptide mimetics that can restore the tumor suppressive function. E.g., ITIH5 [36] is a hyaluronan binding [42] extracellular matrix protein that has been found to confer metastasis suppressive functions in both breast cancer [40] and pancreatic cancer [43]. In the biological therapeutic approach, two different strategies may be followed (Figure 3).

#### 3.1.1. Strategy #1: Small Synthetic Peptides (SSP Strategy)

In order to identify small peptide sequences derived from C2TSG proteins that are able to mediate tumor suppressive functions, an epitope mapping can be applied using hundreds of synthesized peptides of short length (20–40 amino acids) covering the whole C2TSG protein. Flanking peptides should overlap in approximately 85% of sequences to provide detailed coverage of the C2TSG protein. Since synthesized peptides may show varying solubility ranges, different DMSO concentrations might be needed to dissolve these peptides. Subsequently, synthetic peptides will be screened in vitro using cancer cell lines representative of the selected tumor. Cell viability measurements such as CellTiter-Glo (Promega, Madison, WI, USA) or XTT proliferation Kit II (Roche, Basel, Switzerland) are recommended as read-outs, allowing a high-throughput and objective measurement of putative tumor suppressive effects (to discriminate tumor suppressive from, e.g., metabolic effects).

**Figure 4:**
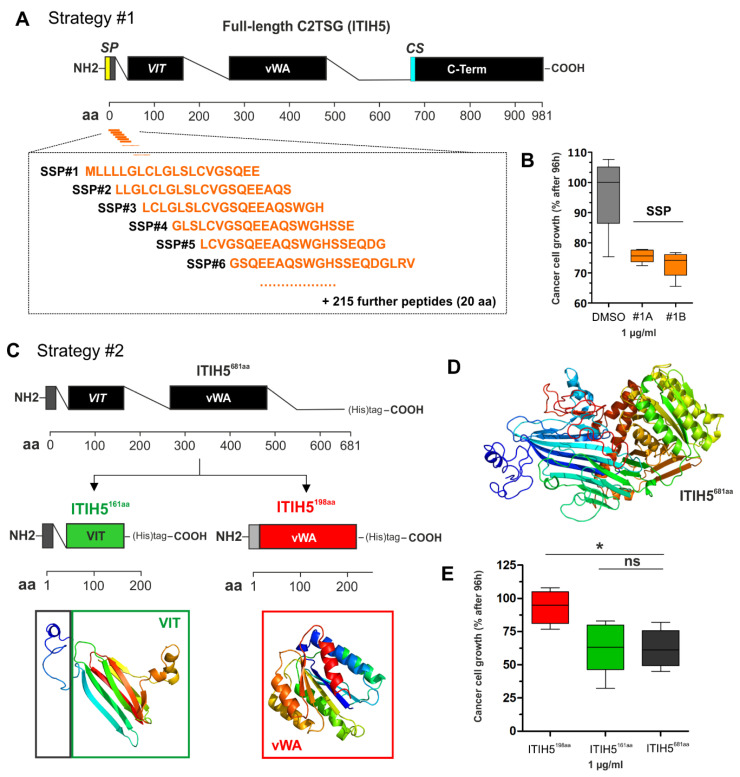
Illustration of the two strategies to engineer mimetics of C2TSG protein based on the ITIH5 example. (**A**) Strategy #1: Schematic cartoon of the full-length ITIH5 protein consisting of 942 amino acids (942 aa), including an illustration of the relative position of synthetic peptides (overall n = 221) covering the N-terminal protein part, i.e., from the signal peptide (SP, aa #1) to the conserved cleavage site (CS, aa #680). VIT: Vault protein inter-alpha-trypsin, vWA: von Willebrand factor, type A, C-term: Inter-alpha-trypsin inhibitor heavy chain, C-terminal. (**B**) Growth of breast cancer cells upon in vitro treatment with peptides #1A and #1B (each 1 µg) compared with the DMSO control using the XTT proliferation assay. (**C**) Strategy #2: Engineering of truncated and recombinant polypeptides either covering the VIT domain (relative aa position according to specific databases: PFAM = 51–159; Prosite profiles = 35–161) (including the endogenous SP) or the vWA domain (relative aa position: PFAM = 295–466; Prosite profiles = 295–478). (**D**) The three-dimensional structure of the N-terminus, the VIT, and vWA polypeptides were predicted and visualized by using Phyre 2 tool [44]. (**E**) Growth of breast cancer cells upon extracellular application of recombinant polypeptides (VIT and vWA, 1 µg) compared with the recombinant N-terminal ITIH5 protein by determining the living cell numbers 96 h after treatment. (ns: not significant, *: *p* < 0.05).

In Figure 4, an example is illustrated for the C2TSG protein ITIH5, where 221 peptides (each with a length of 20 amino acids) were used to cover the N-terminal, i.e., the secreted part of the ITIH5 protein—flanking peptides overlap in 17 of the 20 amino acids, respectively (Figure 4A). Peptides (1 µg) were added to aggressive breast cancer cells (such as MDA-MB-231) for 72 h using the XTT proliferation assays according to the manufacturer’s protocol. Results of reduced cell growth upon peptide treatment in comparison with the DMSO control are shown in Figure 4B.

#### 3.1.2. Strategy #2: Recombinant Truncated Polypeptides (RTP Strategy)

The second strategy (Figure 4C) is recommended if physiochemical and biochemical features of a C2TSG protein are not only dependent on the amino acid sequence (primary structure) but may also be determined by the shape and folding conditions (secondary and tertiary structure). In those cases, a systematic approach of truncated polypeptides derived from the full-length C2TSG protein is required. Polypeptides should orientate towards distinct protein domains of the C2TSG protein according to databases (e.g., PFAM, Prosite, etc.) reflecting putative units of function. Since posttranslational modifications of proteins such as the degree of glycosylation of amino acid residues may be important to ensure full effectiveness, truncated proteins should be recombinantly synthesized in eukaryotic/human in vitro models such as HEK293T (human embryonic kidney cells) or Chinese Hamster Ovary (CHO) cell lines. In addition, a cDNA encoding the truncated C2TSG protein domain should be cloned into an expression vector containing both a kappa leader sequence for effective secretion and a fusion tag tail to allow extraction and purification of the recombinant polypeptides from the supernatant by following the principle of the affinity chromatography. However, the purity of extracted polypeptides must be confirmed for each batch, while enrichment and rebuffering of isolated peptides may be necessary to increase purity.

In Figure 4C–E, the RTP strategy is exemplarily shown for the C2TSG protein ITIH5. Based on the N-terminal, secreted ITIH5 protein (681 aa), two distinct polypeptides were engineered, which comprise either the sequence of the ITIH5 specific VIT (161 aa) or the vWA (198 aa) domain (Figure 4C,D) including a 6× Histidine-tag for purification. Performing functional drug-response analyses in vitro using (aggressive) breast cancer cells upon treatment with the two different recombinant ITIH5 derived polypeptides, we confirmed mimicry of tumor cell growth inhibition mediated by the recombinant VIT protein only. In contrast, the vWA domain polypeptide did not significantly block cancer cell growth. Hence, the recombinant VIT polypeptide may constitute the basis for developing ITIH5 mimetic drugs in the future [45].

##### Preclinical Challenges of the Biological Approach

Various challenges may emerge in the potential therapeutic application, which should be considered already at the early stages of drug development. First—as mentioned before—choosing the right strategy, i.e., either for engineering synthetic or recombinant (poly)peptides, is dependent on the complexity of the underlying mechanisms of the C2TSG protein. A simple and short amino acid sequence fitting to the binding pocket of a protein could be suitable, for instance, to prevent ligand binding, i.e., protein-protein interactions. In turn, since most C2TSG proteins are large polypeptides (>600 amino acids) with complex biochemical and physicochemical features, biotechnology-derived and single functional domain-downsized polypeptides may be required to mimic the heterogenous functional aspects of different structural levels (secondary and tertiary structure) including specific posttranslational modifications.

Small isolated protein domains might be sufficient to mimic the original function of a protein. Protein fragments might even bear clear advantages over full-length proteins for potential patent applications. Despite the various advantages of smaller polypeptides in drug discovery over proteins, they also face a number of pharmacological challenges once they reach the bloodstream. (1) Glomerular filtration clearance is most efficient for proteins smaller than 30 kDa (approximately 270 amino acids), whereas for those exceeding this size, the filtration rate is much lower [46]. For low molecular weight proteins <0.5 kDa, extracellular hydrolysis is the dominating clearance mechanism [46]. (2) Distribution within the vascular space, transport across the microvascular walls, or across cell membranes (also within solid tumors) is size dependent (according to their apparent volume) as well as determined by the physicochemical properties of the polypeptides [47]. (3) Binding of mimetics to circulating plasma proteins can affect both the distribution and clearance of drugs. (4) Uptake of drugs into intracellular components of targeted (cancer) cells unless their mode of action is mediated by extracellular mechanisms. In turn, receptor-mediated uptake by specialized cells such as macrophages followed by intracellular catabolism plays an important role in the total elimination of drugs from the body [46]. Thus, evading the host immune system is a first crucial characteristic of biologicals to avoid degradation, especially of those whose mode of action is mediated from the extracellular site. For (poly)peptides that must be incorporated into cells to be effective, attachment of cell-penetrating peptides (CPP) may help, especially for those macromolecules whose cellular uptake is limited. Accumulating reports have shown that CPP complexes can escape from endocytic organelles and reach the cytosolic space. The release of molecules trapped in endocytic vesicles is essential for intracellular delivery [48]. However, further modifications are crucial to avoid lysosomal degradation.

To overcome those problems, chemical modifications and optimizations of drug candidates are required. For instance, synthetic polymers such as polyethylene glycol are used to reduce clearance rate, however, to the detriment of drug uptake by (cancer) cells [49]. In order to increase (intracellular) stability, drugs could be modified by incorporating non-natural amino acids or pseudo-peptide bonds [50,51]. Still, (poly)peptides are promising tools to engineer anti-cancer drugs mimicking the mechanism(s) of lost C2TSG proteins despite some disadvantages in vivo, in pharmacodynamic and pharmacokinetic studies.

### 3.2. Defining Comprehensive Small Molecule Approaches to Decipher C2TSG Mimetic Drugs

As mentioned in the introduction, many tumors lose the function of important C2TSG due to DNA hypermethylation of their gene promoters. In the small molecule approach, a high throughput screen (HTS) can be performed that identifies novel compounds that can mimic the function of the lost C2TSG protein as precisely and completely as possible. The approach has the goal of providing a spectrum of new potential target structures with associated novel chemistries for those cancers for which there are currently no adequate therapeutic options. Still, a large number of dysregulated C2TSG proteins may be involved. Analyzing the biological and clinical significance of these C2TSG proteins by our presented platform technology will provide a measure of the importance of the involved cancer signaling pathways in the respective cancer entity. It can be expected that the more important the C2TSG protein loss is for tumor initiation and progression in these cancers, the more effective the C2TSG mimetic drugs will be.

## 4. Production of Stable Tumor Cell Lines Expressing C2TSG Proteins

If in silico data from The Cancer Genome Atlas (TCGA) or own tumor cohort expression and/or methylation data (see above) predict that a new C2TSG candidate is likely involved in tumor suppression, corresponding in vitro models are being generated. For that DNA of the candidate C2TSG is transfected into a eukaryotic expression vector in various tumor cell lines, and clones are selected that have stably integrated the C2TSG candidate. In parallel, mock cell clones are generated that have integrated the empty expression vector (without C2TSG candidates), so-called “empty vector” controls. Subsequently, full vector clones (with C2TSG candidate) are compared with empty vector clones in functional cellular assays. Tumor suppressive properties of the C2TSG candidate should become apparent when using various assay read-outs to determine differences between full and empty vector clones in cellular assays, e.g., cell counting, colony formation, apoptosis, and migration [39].

## 5. Importance of Cell Line Biobanking

Well-characterized transfected tumor cell lines, which re-express the C2TSG protein, are being biobanked for long-term storage and accessibility. In our use case SFRP1, the transfected cell lines are shipped from the originating lab to the collaboration partner. The tumor suppressive properties of the C2TSG protein are verified under the cell culture conditions of the partner’s laboratory. After verification, several cell line clones of full and empty vector transfectants are frozen in nitrogen as backup as well as for later quality controls and further use (biobanking).

## 6. Successful Cell-Based Assay for Application of C2TSGs in High-Throughput Screens

**Figure 5:**
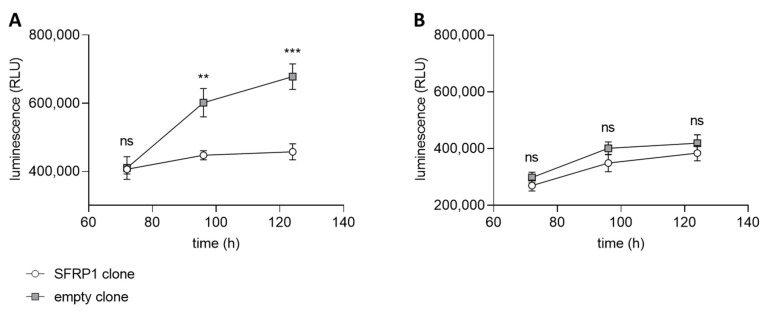
Differential cell viability measurements with SFRP1 as C2TSG. CellTiter-Glo^®^ assay using the breast cancer cell line MCF7 stably transfected with either SFRP1 gene (circle) or empty vector sequence (square). The luminescence (RLU = relative light units) correlates with the number of viable cells at certain time points in the assay. (**A**) Normal breast cancer cell growth of SFRP1 and empty vector clones treated with 0.5% DMSO as solvent control. As expected, the clone expressing SFRP1 shows considerable growth inhibition compared with the empty vector clone. (**B**) SFRP1 and empty vector clones were treated with 50 µM WAY-316606, an SFRP1-specific inhibitor. As expected, the growth behavior of the SFRP1-expressing clone now resembles that of the empty vector clone because the function of the SFRP1 tumor suppressor protein is inhibited. (ns: not significant, **: *p* < 0.01, ***: *p* < 0.001).

For the application of an HTS with a small molecule compound library, an assay based on the transfected tumor cell line with and without the C2TSG protein (full and empty vector) is established in the screening facility. We used the CellTiter-Glo^®^ Luminescent Cell Viability Assay as a read-out for the differential assay. CellTiter-Glo^®^ is a homogenous assay format for the determination of viable cells based on the quantification of available ATP, representing metabolically active cells [52]. As positive and negative controls for the screening assay, we used 10 µM staurosporine (positive control) and 0.1% DMSO (negative control) treated cells. The performance of the screen was controlled by the determination of statistical parameters (signal/noise and signal/background ratios; Z′-values). Only assay plates with Z′-values greater than 0.5 were used for the evaluation. The differential screening model uses both cancer cells, transfected with empty and full vectors, encoding the corresponding C2TSG. Both cell line clones were validated under HTS conditions with the CellTiter-Glo^®^ read-out before screening. In our first HTS approach, we used SFRP1 as C2TSG, expressed in breast cancer cells. SFRP1 is a well-known antagonist of the Wnt-signaling pathway [37], and its loss in various tumor entities has been directly linked to disease progression [53] and a bad prognosis [54]. Both luminal and basal breast cancer cell lines do not express any SFRP1 due to gene promoter hypermethylation [54]. Thus, for the HTS screen, we generated different breast cancer cell lines re-expressing abundant SFRP1. The differential growth of the breast cancer cell line MCF7 independent of SFRP1 and the high specificity of this tumor suppressive function is shown in Figure 5. The growth suppressive function of SFRP1 is validated using WAY-316606, a specific SFRP1 inhibitor. 

MCF7 cells showed considerable growth inhibition in the presence of SFRP1 over time (Figure 5A). When SFRP1-positive cells were treated with the SFRP1-specific inhibitor WAY-316606, growth potential of these cells resembled those of the empty vector clone (Figure 5B). This finding demonstrates that growth inhibition in SFRP1-positive clones is indeed SFRP1-specific and, e.g., not due to a clonal selection bias. Therefore, transfected MCF7 cancer cell lines with and without the C2TSG protein SFRP1 represent a suitable model for a differential HTS with the CellTiter-Glo^®^ assay as a read-out.

## 7. Identifying Mimetic Small Molecules for C2TSG Proteins Using HTS

**Figure 6:**
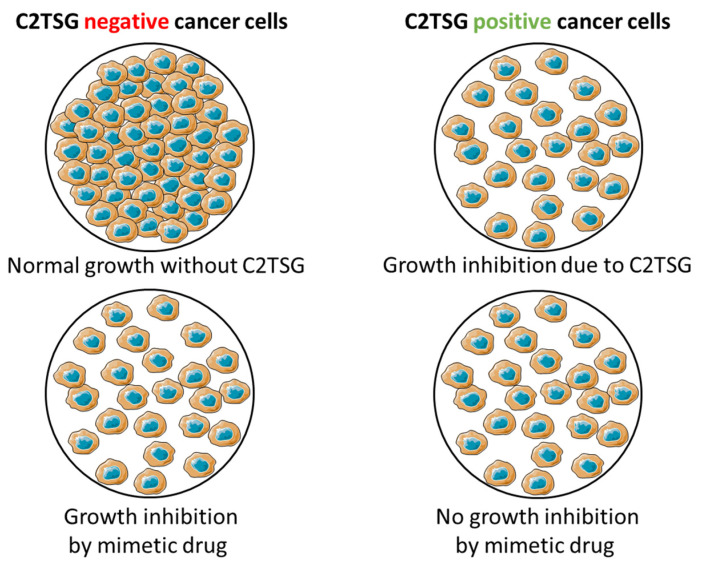
General principle of screening for mimetic drug candidates based on the CellTiter-Glo^®^ Assay. Cancer cell line lacking C2TSG protein exhibits normal growth characteristics (**top left**), while cancer cell line with forced C2TSG protein re-expression exhibits growth inhibition (**top right**). A compound acting like a mimetic drug in C2TSG protein-negative cancer cells will have a growth inhibitory effect (**bottom left**), while the same mimetic drug will have no effect in C2TSG positive cancer cells (**bottom right**). These two aspects, taken together, make a suitable candidate for a mimetic drug candidate.

After establishing and characterizing both MCF7 transfected cell lines, lacking (empty clone) and expressing (full clone) the C2TSG protein of interest (SFRP1), assay development for automation during HTS has been initiated, employing both cell lines in a differential HTS campaign to identify potential mimetic hits mimicking SFRP1 function. The underlying principle of the screening strategy is schematically illustrated in Figure 6 and shows how suitable mimetic hits are selected. A suitable mimetic hit is a compound that inhibits cancer cell growth in the C2TSG protein-negative cells, while in cancer cells with C2TSG protein, it does not show any further growth inhibition besides the mediated growth inhibition by the C2TSG protein itself. These two key features (inhibition of C2TSG non-expressing vs. no inhibition of C2TSG expressing cell lines) taken together characterize a “mimetic” hit for which the extent of the growth inhibitory effect is an additional selection criterion. Somewhat simplified, the stronger the cell growth inhibitory effect in C2TSG non-expressing cell lines, the better the potential of the mimetic drug candidate.

Finally, we performed an HTS of 189,250 compounds in our C2TSG case study, using the Wnt antagonist SFRP1. The primary screen was conducted at a single compound concentration (10 µM) using Cell Titer-Glo^®^ as a read-out. This screen led to the identification of 430 compounds as primary hits based on a pre-defined cut-off for the differential cell growth between the SFRP1 negative and positive cancer cells. These compounds are the first hits, representing potential SFRP1 mimetic drug candidates. For hit validation and prioritization of these compounds, we determined their half maximal inhibitory concentration (IC_50_) on the growth inhibition of the C2TSG negative and positive cancer cells. By setting a certain cut-off value, we reduced the number of verified hit compounds to 96 and also introduced structural prioritization based on the chemical structures of these hit compounds. These 96 compounds were considered as confirmed SFRP1 mimetics for further validation in secondary assays.

## 8. Secondary and Orthogonal Assays Are Key to Further Prioritize Mimetic Drug Candidates Derived from HTS

**Figure 7:**
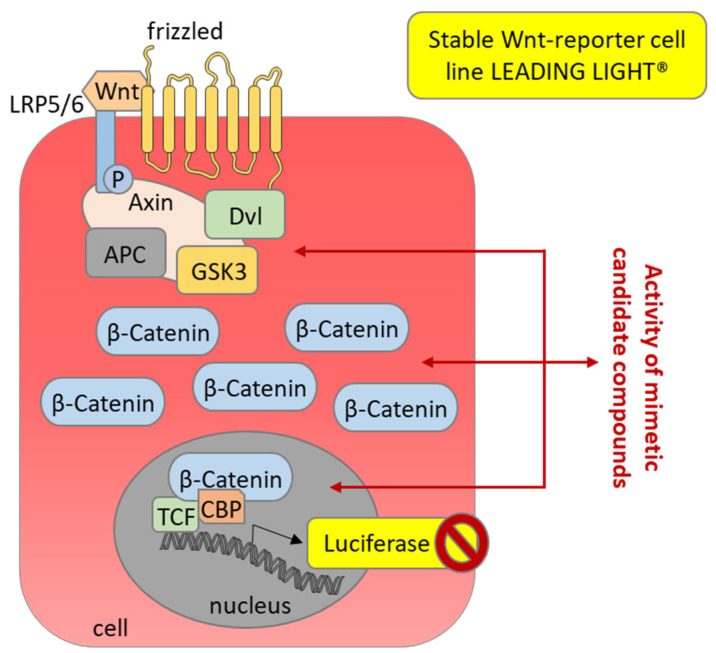
Secondary assay for the primary SFRP1 mimetic hits analyzing activity as Wnt pathway antagonist. The Wnt-reporter cell line LEADING LIGHT^®^ (Enzo Life Sciences, Farmingdale, NY, USA) expresses luciferase upon stimulation of the Wnt pathway after the addition of recombinant Wnt3a as a Wnt signaling ligand. Candidate SFRP1 mimetic hits might inhibit the Wnt signaling pathway at different levels, which should result in inhibition of the luciferase-induced luminescence.

In a case study of SFRP1 as the C2TSG protein, the Wnt signaling pathway plays a crucial role. Therefore, the Leading Light^®^ Wnt Reporter Gene Assay was used as a secondary assay. This assay is based on an engineered 3T3 mouse fibroblast cell line that expresses the firefly luciferase reporter gene independent of Wnt-responsive promoters (TCF/LEF). Using this secondary assay, the SFRP1 mimetic primary hits were prioritized according to their activity in inhibiting the Wnt signaling pathway. SFRP1 mimetic compounds should functionally act like the native protein SFRP1, leading to a decreased luminescent signal in this assay. Using this assay, the 96 primary SFRP1 mimetic hits from HTS were reduced to 59 compounds (Figure 7), which all inhibited Wnt3a-induced signal transduction and luciferase expression.

**Figure 8:**
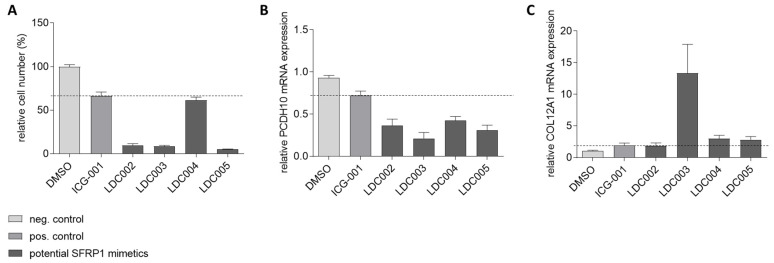
Secondary target gene assay in SFRP1-negative MCF7 breast cancer cells. Analysis of four LDC compounds (LDC002-005) and the reference inhibitor ICG-001 is shown as an example. (**A**) Relative cell growth in the presence of each analyzed compound is lower than for the positive control (ICG-001, dotted line). (**B**) Relative mRNA expression of *PCDH10*, a gene downregulated by SFRP1, induced by the analyzed compounds is below the cut-off of the positive control (ICG-001, dotted line). (**C**) Relative mRNA expression of *COL12A1*, a gene upregulated by SFRP1, induced by the analyzed compounds is at least as high or above the cut-off of the positive control (ICG-001, dotted line). Remarkably. LDC003 shows a highly significant *COL12A1*-induction effect.

An mRNA target gene analysis for native SFRP1 was performed to identify target genes that are specifically regulated by SFRP1. These SFRP1-regulated candidate genes had been previously determined in the empty and full SFRP1 vector clones by array-based expression profiling. One specifically downregulated gene (*PCDH10*), and one specifically upregulated gene (*COL12A1*) were chosen as marker genes for hit validation, to be used as cellular pharmacodynamic (PD) RNA markers as an additional secondary assay format, probing for SFRP1-like functional cellular activity of the confirmed screening hits. For the secondary assay, the empty vector-transfected breast cancer cells were used to investigate the potential effects of the SFRP1 mimetic hits on target gene regulation. Compounds that specifically down-regulate *PCDH10* while up-regulating *COL12A1*—just like SFRP1—and have a clear growth inhibitory effect on cells were further selected as potential SFRP1 mimetic hits for medicinal chemistry-based optimization. This 3-factor selection was controlled by introducing the well-characterized Wnt pathway inhibitor ICG-001 [55] as a reference. DMSO is used as a negative control. Figure 8 does show four representative hits from different chemical series that have been tested for expression of *PCDH10* and *COL12A1*. While all hit compounds (LDC002-LDC005) and the ICG-001 reference show reduced *PCDH10* expression, upregulation of *COL12A1* has been observed only for LDC003, LDC004, and LDC005 (Figure 8).

**Figure 9:**
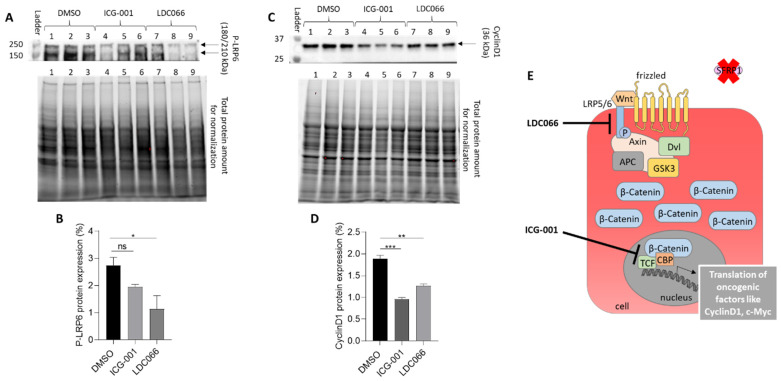
Secondary pathway assay for a representative and optimized SFRP1 mimetic hit, showing potential regulation of the Wnt/β-catenin signaling in BT20 breast cancer cells. DMSO (lanes 1, 2, 3) serves as negative diluent control and ICG-001 (lanes 4, 5, 6) as reference drug. (**A**,**B**) Protein expression of the phosphorylated LRP6 receptor (P-LRP6) shows significant down-regulation in the presence of LDC066 (LDC066 is an optimized analog of an SFRP1 mimetic screening hit, lanes 7, 8, 9), but not by ICG-001. All compounds are added as triplicates (ICG-001 at 20 µM, LDC066 at 10 µM). (**C**,**D**) Protein expression of the transcription factor cyclin D1 is significantly downregulated by ICG-001 and LDC066 since both substances down-regulate the Wnt signaling cascade at the level of gene transcription, and cyclin D1 is a TCF/CBP-regulated gene. (**E**) Cartoon of active Wnt/β-catenin signaling cascade. LDC066 seems to down-regulate the active LRP6 receptor (“P-LRP6”) at the cell surface, while the reference drug ICG-001 antagonizes the TCF/β-catenin/CBP-dependent transcription in the cell nucleus. Consequently, both drugs mediate down-regulation of the oncogenic transcription factor cyclin D1, a key transcription factor in active Wnt signaling. (ns: not significant, *: *p* < 0.05, **: *p* < 0.01, ***: *p* < 0.001).

Using this small molecule and “black box” screening approach, the molecular binding site of any SFRP1 mimetic hit initially remains unknown unless there is a striking chemical similarity if not identity with known molecular targets in the Wnt3a signaling pathway. Therefore, the identification of the molecular receptors of the mimetic hit(s) is a crucial and mandatory step in the further development of any drug candidate towards clinical development. In the case study for SFRP1 mimetic hits, we investigated if any of the optimized hits (after several rounds of medicinal chemistry) is able to modify protein amounts or phosphorylation patterns of specific molecules in the oncogenic Wnt/β-catenin signaling pathway (Figure 9).

The novel and partially optimized SFRP1 mimetic lead, LDC066, seems to specifically down-regulate the phosphorylated LRP6 receptor (P-LRP6) at the cell surface, while the reference drug ICG-001 shows no significant effect on P-LRP6. ICG-001 is known to bind directly to the TCF/β-catenin/CBP-complex [55] and thus leading to the inactivation of Wnt signaling by down-regulation of the oncogenic transcription factor cyclin D1. Since perturbations of the Wnt signaling pathway at different steps from the cell membrane to the nucleus converge in suppressing the TCF/β-catenin/CBP transcriptional complex, both ICG-001 and the SFRP1 mimetic candidate LDC066, resulting in cyclin D1 down-regulation, a key transcription factor in active Wnt signaling.

These data are considered proof-of-concept for an efficient screening and hit selection process using a novel differential cellular screening paradigm to mimic the activity of C2TSG proteins. With this novel paradigm, we hope to add to the druggability of tumor suppressors in general and C2TSGs in particular. We consider the SFRP1 differential screen a blueprint for future C2TSG screening campaigns, which ultimately might significantly broaden the spectrum of potential anti-cancer drug candidates and drugs. Hit-to-lead optimization is currently underway and is based on chemical modification of the original hit compounds from different compound classes. Optimization parameters cover increased cell-based activity as well as in vitro and in vivo DMPK (Drug Metabolism and PharmacoKinetic) parameters. Selected improved compounds will then be tested in vivo in mouse models for Wnt signaling and tumor inhibition in parallel with pharmacodynamic parameters. This case study of highly specific SFRP1 mimetics with antiproliferative properties demonstrates the potential of this new therapeutic platform in personalized cancer medicine.

## 9. Outlook

### 9.1. The Age of Personalized Cancer Therapy Is Still Young

Personalized targeted therapies have been and still are revolutionizing how we treat cancer patients. The switch from originally organ-centric and chemotherapy-based concepts guiding cancer treatment towards a deep molecular analysis of the affected tumor tissue to identify actionable alterations or “drivers” has personalized tumor treatment on the individual scale. Central, of course, is the question of to what extent this personalization of cancer therapy has become linkable with significantly improved survival of the affected patients. The answer varies considerably according to different cancer entities but also according to sub-entities of a given cancer type. In lung cancer, for example, cancer-specific survival improved from 26% to 35% for NSCLC patients diagnosed in 2001 versus 2014 [56]. A major contribution to this impressive improvement in survival was probably the increasing use of first-line tyrosine kinase inhibitors such as Erlotinib and Gefitinib [57,58]. Fortunately, this beneficial response was found across both sexes, all races, and ethnic groups. In contrast, there were limited treatment advances for SCLC in this time frame [56]. And for example, in breast cancer, the annual decrease in mortality of nearly 2% in the last 20 years has been mainly attributed to mammography screening and better chemotherapy and not personalized therapies, such as endocrine therapy or trastuzumab [59]. Thus, there is still a lot of space to improve personalized cancer treatment in the future. Important new routes are definitively the rising fields of immune-oncology [60], cancer gene therapy [61], personalized cancer nanomedicines [62], and potentially C2TSG protein mimetics, as suggested in this white paper.

### 9.2. Which Tumor Entities May Be the Best Entry Points for Personalized Cancer Therapies Using Mimetic Drugs Based on C2TSG Proteins?

It is obvious that cancer therapies based on new concepts should first be tested in model systems of such tumor entities, where our current approaches (including targeted therapies based on genetic driver alterations) have not yet been sufficiently successful. Looking at the year 2021, estimated numbers of new cancer cases (N) and estimated deaths (D) for each common cancer type as summarized by the National Cancer Institute website cancer.gov (accessed on 14 June 2022) [63], pancreatic cancer (60,430 N, 48,220 D) and liver/bile duct cancer (42,230 N, 30,230 D) are difficult to treat entities, but certainly with a high demand for new therapeutic approaches. These three tumor entities are also known as hepato-pancreato-biliary (HPB) cancers [64] and are complex to treat since HPB surgery is associated with major morbidity and significant mortality [65]. A putative C2TSG that has recently been identified in liver cancer is the well-known (mutated) tumor suppressor gene *ARID1A*. While a relationship between *ARID1A* promoter hypermethylation and disease progression had been shown before for squamous cell carcinoma [66], the situation was unclear for hepatocellular carcinoma (HCC). However, a recent study analyzing the TCGA data set could show that low *ARID1A* expression in HCC is clearly associated with poor prognosis. This loss of ARID1A expression is likely primarily due to promoter DNA hypermethylation [67] and not to *ARID1A* mutations, which also occur in HCC, but only with a frequency of 4–17% [68]. Having identified ARID1A as an interesting C2TSG protein in different tumor entities, what would be the best strategy to obtain suitable mimetic drugs to substitute for the tumor lost ARID1A protein? The small molecule approach or the recombinant truncated polypeptide approach? The ARID1A protein is a very large polypeptide consisting of 2285 amino acids with multiple domains and isoforms and an even more complex context-dependent biology [69]. Within such a large protein, it could prove quite difficult to define and narrow down the polypeptide regions relevant for tumor suppression without losing co-ruling regions. Thus, a small molecule approach based on an HCC tumor cell line that has lost ARID1A through promoter DNA hypermethylation combined with a phenotypic differential screen for candidate ARID1A mimetics may be the better approach, by far. The polypeptide mimetics approach, using, e.g., recombinant truncated polypeptides, probably may have advantages if the C2TSG encodes a secreted protein with proven extracellular functions.

Pancreatic cancer is another highly lethal disease for which mortality closely parallels incidence [70], and the major driver gene KRAS is currently not druggable except for some early responses with Sotorasib treatment in those 1–2% of patients carrying the KRAS p.G12C mutation [71]. Still, Sotorasib is considered a breakthrough in KRAS-dependent cancer therapy. Both of the C2TSG proteins presented above, i.e., SFRP1 and ITIH5, are also downregulated due to promoter hypermethylation in pancreatic cancer (our unpublished data) and recent studies in an animal model for metastasis (ITIH5) [43] and a clinical model for drug response (SFRP1) [72] have indicated the importance of these C2TSG proteins in suppressing pancreatic cancer progression.

### 9.3. Molecular Companion Diagnostics to Decipher Patients Suitable for Therapy with C2TSG Protein Mimetics

The widespread availability and continued advancement of next-generation sequencing (NGS) technology have spurred driver gene-based molecular tumor diagnostics and thus therapy [73]. Entirely analog, the precise and accurate determination of a gene’s promoter DNA methylation status in the tumor tissue will be crucial to decide whether C2TSG protein mimetics may be applicable with therapeutic success for a given patient. Thus, personalized cancer medicine based on C2TSG mimetic drugs will need companion diagnostics based on robust technologies. Here, two state-of-the-art procedures are available that have similar skill and budget requirements as NGS technology. Droplet digital (ddPCR) is a recent advance in PCR technology that enables the precise detection and absolute quantification of nucleic acid target sequences. It can easily be adapted for accurate determination of the promoter DNA methylation status of a C2TSG [74]. Pyrosequencing, which is a DNA sequencing technology based on the sequencing-by-synthesis principle, can also be applied successfully to measure the amount of promoter DNA methylation in C2TSG [75]. In short, the analysis of DNA methylation patterns by pyrosequencing combines a simple reaction protocol that measures the degree of methylation of several CpGs in close proximity with high quantitative resolution [76]. Quantitative epigenotypes are obtained using this protocol in approximately 4 h for up to 96 DNA samples when bisulfite-treated DNA is available as starting material. In summary, the technological prerequisites for companion diagnostics that enable the selection of potential responders for C2TSG protein mimetics are already in place.

## 10. Summary of the Key Statements of This White Paper

- Restoring the lost function of tumor suppressor genes (TSGs), especially of class 2 tumor suppressor genes (C2TSGs), might be a valid approach to treating cancer, with an initial focus on those tumor entities where current personalized medicine approaches have not spawned “game-changing” therapies so far.
- The restoration of TSGs, the defective “brakes in the car-of-life,” by means of gene therapy or through the use of DNA demethylating agents and histone deacetylase inhibitors have not been particularly successful over the last two decades.
- Thus, new strategies are needed to exploit the therapeutic potential of C2TSGs or DNA methylation cancer driver genes (“DNAme drivers”), which, thanks to new technologies, can also progressively been better validated.
- A concept for a new and still early-stage therapeutic approach is presented, focusing on the phenotypic imitation (“mimesis”) of proteins encoded by highly disease-relevant C2TSGs/DNAme drivers.
- In the biological approach, mimetic drug candidates are based on truncated and/or genetically engineered C2TSG proteins, consisting solely of domains with defined tumor suppressive function.
- In the small molecule approach, mimetic drug candidates are derived from a differential HTS using cell-based assays comparing C2TSG expressing and C2TSG devoid cancer cells.
- Molecular companion diagnostics to identify patients suitable for therapy with C2TSG protein mimetics should be readily developable, similar to NGS-based cancer driver diagnostics.

## 11. Conclusions

Restoring the lost function of tumor suppressor genes (TSGs), especially class 2 tumor suppressor genes (C2TSGs), could be an effective approach treating cancer. Currently, it is too early to expect such exploratory C2TSG protein mimetics projects in clinical trials, but once they do, it will greatly inspire and advance the field, as the potential of reactivatable C2TSG protein “targets” in any cancer entity is huge. We hope that our concept will be lively discussed and that the White paper will help to drive research and development in this direction.

## Data Availability

The data supporting this White paper's findings are available from the authors upon reasonable request.
